# The many faces of *Candida auris*: Phenotypic and strain variation in an emerging pathogen

**DOI:** 10.1371/journal.ppat.1012011

**Published:** 2024-03-01

**Authors:** Darian J. Santana, Guolei Zhao, Teresa R. O’Meara

**Affiliations:** 1 Department of Microbiology and Immunology, University of Michigan Medical School, Ann Arbor, Michigan, United States of America; 2 Department of Epidemiology, University of Michigan School of Public Health, Ann Arbor, Michigan, United States of America; University of Georgia, UNITED STATES

## Abstract

*Candida auris* is an emerging fungal pathogen with unusual evolutionary history—there are multiple distinct phylogeographic clades showing a near simultaneous transition from a currently unknown reservoir to nosocomial pathogen. Each of these clades has experienced different selective pressures over time, likely resulting in selection for genotypes with differential fitness or phenotypic consequences when introduced to new environments. We also observe diversification within clades, providing additional opportunities for phenotypic differences. These differences can have large impacts on pathogenic potential, drug resistance profile, evolutionary trajectory, and transmissibility. In recent years, there have been significant advances in our understanding of strain-specific behavior in other microbes, including bacterial and fungal pathogens, and we have an opportunity to take this strain variation into account when describing aspects of *C*. *auris* biology. Here, we critically review the literature to gain insight into differences at both the strain and clade levels in *C*. *auris*, focusing on phenotypes associated with clinical disease or transmission. Our goal is to integrate clinical and epidemiological perspectives with molecular perspectives in a way that would be valuable for both audiences. Identifying differences between strains and understanding which phenotypes are strain specific will be crucial for understanding this emerging pathogen, and an important caveat when describing the analysis of a singular isolate.

## Introduction

Fungal diseases account for over 6.5 million invasive infections annually, with nearly one quarter of these attributed to members of the *Candida* genus [1]. In 2009, a clinical report detailed the isolation of a previously uncharacterized pathogenic member of this genus, *Candida auris*, from the ear canal of an inpatient in a Japanese hospital [2]. This report marked the beginning of the emergence of a globally distributed, often multidrug-resistant, outbreak-capable pathogen, ultimately recognized by the United States Centers for Disease Control and Prevention (CDC) and the World Health Organization as an urgent and critical public health threat [2,3]. Within a decade of its initial characterization, surveillance initiatives defined the nearly simultaneous emergence from a currently unknown reservoir of distinct *C*. *auris* genetic lineages in dozens of countries across all 6 major continents [4,5], driven both by multiple geographic origins and carriage through patient travel [5]. *C*. *auris* infection presents similarly to candidiasis caused by other *Candida* species, with the most severe cases attributed to candidemia and subsequent organ dissemination [6]. Unlike related *Candida* species, however, *C*. *auris* is frequently reported in association with nosocomial transmission, leading to clonally disseminated outbreaks in healthcare settings and in some circumstances, multiyear and regionally endemic spread [7,8]. Surveillance efforts in the US and Europe have recognized exponentially increasing rates of outbreaks since the introduction of *C*. *auris* into these regions, highlighting the difficulty in containing this organism [9,10]. In outbreak settings, *C*. *auris* persistently colonizes patient skin, hospital surfaces, and medical devices, demonstrates contaminative and fomite transmission between individuals, and causes invasive infections, often with high rates of mortality and widespread acquired antifungal resistance [5,8,11–15]. For these reasons, *C*. *auris* is recognized as a critical public health threat and represents a substantial clinical and infection prevention challenge.

Four major genetic lineages of *C*. *auris* have been extensively described, with origins clustering geographically in South Asian (I), East Asian (II), African (III), and South American (IV) clades [5]. Sparse reports of isolates that are genetically distinct from the 4 major clades suggest at least 2 additional lineages, with isolates having geographic links to Iran (V) or Singapore and Bangladesh (VI) [16–18]. Notably, the clades are genetically well separated, differing by tens of thousands to hundreds of thousands of single nucleotide polymorphisms (SNPs) [18–20]. Even within clades, individual strains can differ by thousands of SNPs, exhibit karyotypic diversification, and have stable chromosomal rearrangements [5,19–22]. Increasing evidence even suggests isolates collected from clonal outbreaks, differing only by small numbers of SNPs, can exhibit clinically meaningful phenotypic variation, suggesting the possibility of adaptation within the timescale of outbreak settings [23–25]. The result is the emergence of at least 6 highly divergent genetic lineages of *C*. *auris* and within-lineage variation, ultimately associated with divergent clinically relevant phenotypes such as antifungal resistance [5], virulence and pathogenesis in infection models [26–28], body site tropism [16,29], outbreak potential [29,30], morphogenesis [31], host colonization [32], disinfection resistance [33–35], and metabolite utilization [36].

In this review, we explore experimental, surveillance, and clinical data to synthesize evidence of clinically impactful variation between strains and between clades of *C*. *auris*. We perform a systematic analysis of the most extensively described examples of variation and propose mechanistic models to understand the basis and scope of such variation and to clarify ambiguity present in isolated reports. Finally, we offer hypotheses to promote further research pertaining to the mechanistic and molecular bases for medically relevant behavior in *C*. *auris* and perspectives around studying a global, emerging, divergent pathogen.

### Topical focus

We performed a systematic search of PubMed, Web of Science, and Scopus databases with the only search term “Candida auris” to identify all records from inception until August 4, 2023. Search results were deduplicated using the Systematic Review Accelerator [37] to yield 1,945 unique reports. Titles and abstracts for all reports were reviewed, and each study was categorized thematically. A weekly recurring automated PubMed search for “Candida auris” was performed to identify and categorize new reports as appropriate during manuscript preparation. Based on categorical representation, topics were selected that were most likely to encompass characterization of clinically relevant strain variation.

Among clinical reports, 192 included more than 1 patient. The full text of each of these reports was reviewed, and data were systematically extracted using a standardized form to record relevant findings. To perform a meta-analysis of crude mortality, 39 reports [4,6,8,14,38–76] were selected that met the acceptance criteria of having (1) At least 5 infection cases; (2) Determination of clade made by the authors or identifiable through publicly available analyses through one or more molecular typing techniques; and (3) Crude in-hospital mortality reported. Meta-analysis with subgroup analysis was performed using the meta R package (version 6.5–0) using a random intercept logistic regression model with logit-transformation, maximum-likelihood estimator for τ^2^ without a common estimate across subgroups, and the Clopper–Pearson confidence interval for individual studies with a continuity correction of 0.5 for studies with zero cell frequencies.

For experimental virulence models, 11 reports of invertebrate or murine infection models [24,25,28,77–84] were identified that met the acceptance criteria of having (1) Multiple isolates with clade or body site origin indicated; (2) Identical infection protocol between isolates; and (3) Survival data available. Because of the small number of studies available, an acceptance criteria including any study that compared more than 1 isolate from different groups was established, with the anticipated limitation that studies with small sample sizes might exhibit greater variation from a true effect. For each study, isolates were ranked by virulence score: first by overall mortality (number of mortality events or time to 100% mortality, whichever was more appropriate) then by median survival time. Survival results for individual isolates were plotted by within-study ranks, colored by clade or origin.

To evaluate outbreak sizes, 109 clinical reports were selected that met the acceptance criteria of having (1) At least 2 linked cases; (2) Single center or clustered multicenter outbreak; (3) Dates of the outbreak or data collection reported; and (4) Location of the outbreak reported. Outbreak size was determined as the total number of affected patients (colonized or infected), and average rate was determined by dividing the total number of affected patients by the length of time sampling was performed.

## *C*. *auris* association with human hosts

While limited evidence supports the possibility of environmental, zoonotic, or foodborne reservoirs of *C*. *auris* [85–90], the best understood reservoir for carriage, transmission, and dispersal is the human body. *C*. *auris* exhibits both asymptomatic and infectious associations with susceptible hosts, and understanding the dynamics of these associations has been a topical focus for much of the clinical and experimental literature. Critically, decontamination of colonized or infected patients with antiseptics or antifungals has presented substantial clinical challenges, and recent work has highlighted some of the biological underpinnings exacerbating these difficulties.

### Virulence

*C*. *auris* persistently colonizes multiple body sites. Most prominently, skin sites such as the nares, palms, fingertips, axillae, inguinal creases, and toe webs show high positivity, but asymptomatic isolation from other nonsterile body sites such as lungs and urine is not uncommon [11,91]. Unlike for some other human-associated *Candida* species, colonization is likely a rare event specific to individuals with healthcare exposures. For instance, a recent search of approximately 300,000 publicly available metagenomic runs found only 20 runs from 5 projects likely containing *C*. *auris* genomic information, most of which were specifically linked to surveillance initiatives in *C*. *auris* outbreak settings [92]. Colonization can increase the risk for disseminated infection, especially candidemia [93,94]. One report found that approximately 5% to 10% of colonized patients develop bloodstream infections [58], while another estimated a 25% cumulative risk of candidemia 60 days after initial detection of colonization [42]. In addition, urinary tract infections, wound infections, otitis, and skin abscesses are common, but *C*. *auris* is not prominently associated with other common types of candidiasis such as oral thrush or vulvovaginal candidiasis [95]. The clinical presentation of invasive disease is often nonspecific and indistinguishable from other types of systemic microbial infection, and reported in-hospital crude mortality rates for *C*. *auris* infection range from 25% to 70% [96,97].

**Fig 1:**
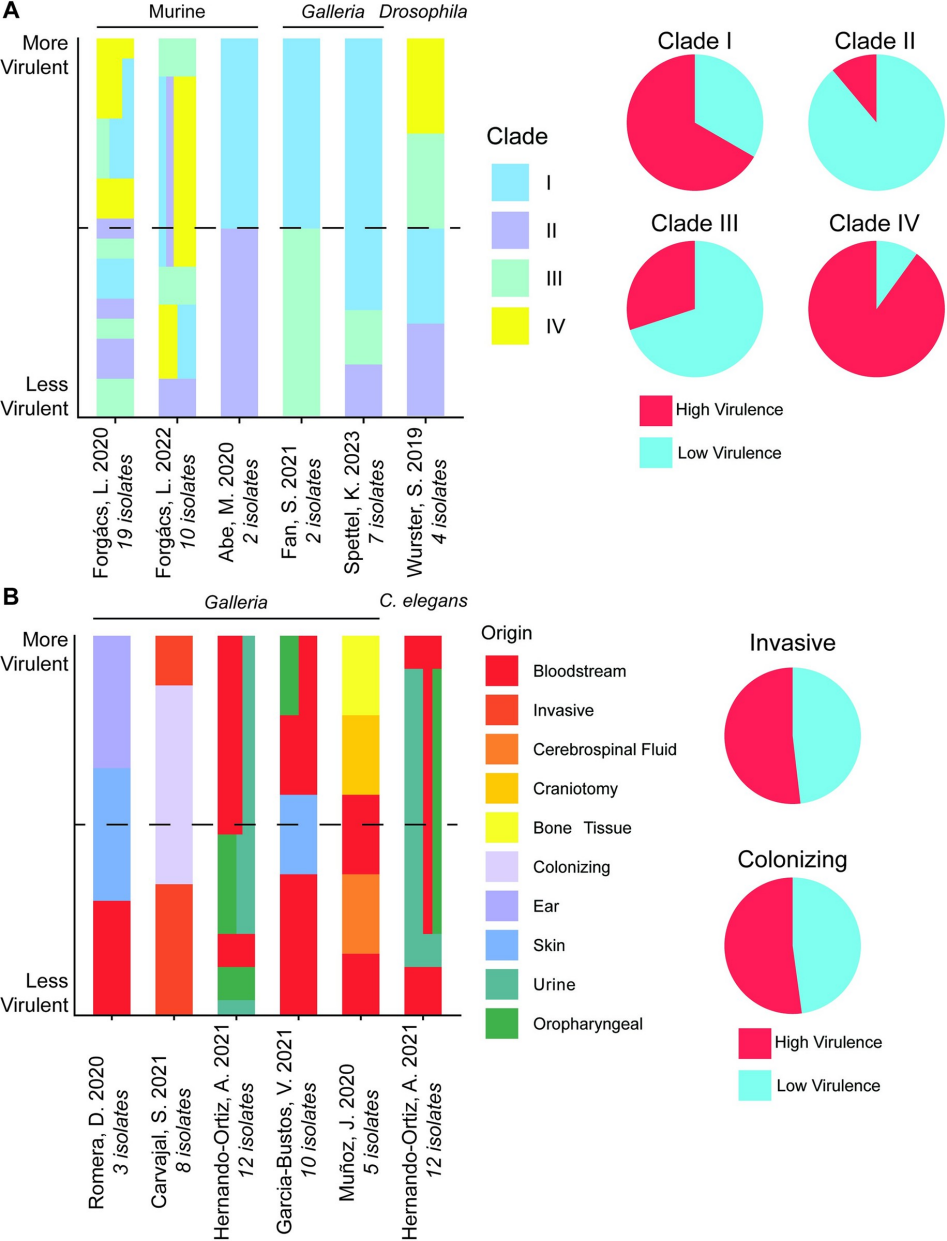
Experimental infection models suggest *C*. *auris* exhibits clade-specific but not body site specific virulence. Survival data were extracted from studies [24,25,28,77–84] directly comparing virulence between isolates of different clades (**A**) or body site origins (**B**) in animal infection models. For each study, the virulence of each isolate was ranked by mortality and median survival. Stacked bars plot of each isolate per study, with higher virulence rankings plotted at the top. The horizontal dashed line corresponds to the average virulence rank for each study. Because isolates were ranked on survival by time to mortality and median survival time, isolates with tied ranks are plotted at the same height on the y-axis. Where isolates from multiple groups share a tied rank, the width of bars grouped horizontally corresponds to the proportion of isolates of that rank attributable to a given group. Pie charts summarize the survival rankings by bisecting all isolates into high or low virulence scores, corresponding to a virulence rank greater than the median of within study comparators (High Virulence) or less than the median (Low Virulence). For panel B, hot colors indicate invasive origins and cool colors indicate colonizing origins.

Multiple mammalian and invertebrate infection models have been employed to characterize *C*. *auris* virulence, with some reports directly comparing isolates of distinct genetic lineages or origins [24,25,27,28,77–84]. Independently, 2 separate reports concluded that isolates from clade IV showed the highest virulence of the 4 major clades in either murine or silkworm infection models [27,28]. We found this to be consistent across multiple reports, with clade IV and clade I isolates generally exhibiting virulence greater than the median of all comparators and clade II and clade III generally exhibiting virulence below the median (Fig 1A). Notably, of the 4 major clades, only clade II is not associated with human-invasive infection or outbreaks, consistent with its poorer pathogenicity in infection models [27–30]. One possible mechanism of this phenotypic diversity is adaptation in response to host association. Based on reports comparing the virulence of isolates recovered from different body sites, however, we found no evidence of differential pathogenicity associated with strains originating from either invasive or colonizing sites (Fig 1B). Together, this analysis suggests genetic lineage, but not within host adaptation, predicts *C*. *auris* virulence in infection models.

**Fig 2:**
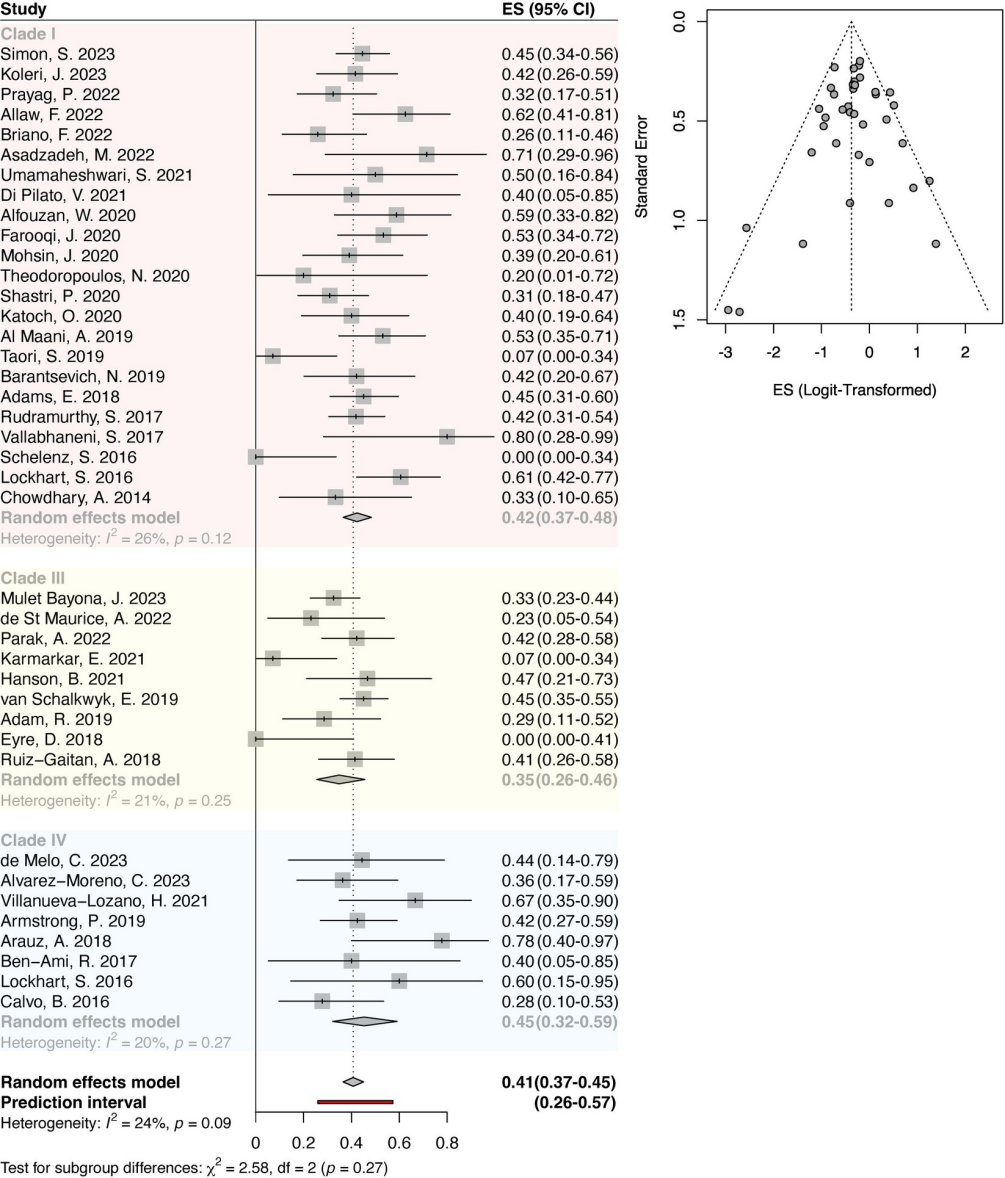
Forest plot on crude mortality of *C*. *auris* infection reports shows nonsignificant variation in crude mortality rates by clade. Effect size (crude mortality) and 95% confidence interval are reported for each included report [4,6,8,14,38–76]. A random effects model was used to estimate overall crude mortality (vertical dotted line included for reference across each report) and the average crude mortality for each clade through subgroup analysis. Funnel plot included for investigation of evidence of publication bias.

To investigate whether the clade-specific virulence was reflected in human infection, we performed a meta-analysis of crude mortality after *C*. *auris* infection for all case reports comprising at least 5 infected individuals and for which *C*. *auris* clade was reported based on molecular typing. As clades II, V, and VI have not yet been associated with outbreaks, no case reports met the selection criteria for number of infected individuals. For the remaining reports comprising isolates from clades I, III, and IV, pooled crude mortality was 41% (95% CI: 37% to 45%) with a prediction interval of 26% to 57%, which aligns well with previous reports of crude mortality for *C*. *auris* infection (Fig 2). Egger’s test gave a *p*-value of 0.4095, indicating no evidence of publication bias (Fig 2), and heterogeneity among reports was marginal and nonsignificant (I^2^ = 24%, *p* = 0.09). Subgroup analysis indicated nonsignificant differences between *C*. *auris* clades (χ^2^ = 2.58, df = 2, *p* = 0.27). However, clade IV showed the highest effect size (45% mortality, 95% CI: 32% to 59%), followed by clade I (42% mortality, 95% CI: 37% to 48%), then clade III (35% mortality, 95% CI: 26% to 46%). While these differences do not reach statistical significance, the trend agrees with the attributable mortality data demonstrated by the experimental virulence models, suggesting clade IV and potentially clade I may truly exhibit greater pathogenicity during infection. Importantly, our meta-analysis only included crude mortality and so is likely underpowered to describe attributable differences by *C*. *auris* clade. Moreover, because the genetic lineages have historically clustered geographically, it is difficult to uncouple strain-specific virulence from regionally differential healthcare practices. However, our analysis suggests the possibility that clade IV is more virulent than other clades.

### Host colonization

In vitro and in vivo models have been developed to study skin colonization by *C*. *auris*. In an in vitro model using an artificial human axillary sweat medium and ex vivo porcine skin as a substrate, *C*. *auris* was able to produce dense, multilayered accumulation [98]. Interestingly, *C*. *auris* exhibited a fungal burden 10-fold greater than that of *C*. *albicans* in the artificial sweat medium but not RPMI-MOPS, along with an apparent persistence of *C*. *auris* in concentrated sweat medium designed to mimic the evaporation of sweat even after 14 days, where the *C*. *albicans* cells were not viable [98]. This finding suggests a distinct capacity for growth in a physiological environment that might be encountered during colonization of high-sweat body sites such as the axillae or inguinal crease. The salt concentration in this synthetic sweat medium is around 3%, and other reports have found this level of salinity to be well tolerated *by C*. *albicans*, suggesting the differential growth capacities in this media between *C*. *albicans* and *C*. *auris* may not be attributed to differences in halotolerance alone, despite increased halotolerance in *C*. *auris* compared to other clinically relevant *Candida* species [99,100]. In this same model, the observed substantial fungal burden was achieved by clinical isolates of all 4 major clades of *C*. *auris* without notable differences [101]. This behavior likely reflects an advantage for *C*. *auris* in human skin colonization, which serves as a source for nosocomial transmission between patients. Notably, in this model, fungal burden was extensive on the skin surface, but there was no evidence of invasive growth into the dermis.

Another study, using an in vivo murine model of skin colonization, observed varying levels of fungal burden following colonization with individual isolates from each of the 4 major clades [32]. In this model, *C*. *auris* was applied onto the shaved mouse skin. A clade III isolate showed the greatest fungal burden 14 days after infection, followed by clade IV, then clade I, and a clade II isolate exhibited the lowest fungal burden. Notably, histopathological evidence suggested *C*. *auris* cells invaded deeper into the skin tissue and resided within the hair follicle [32]. Furthermore, *C*. *auris* cells were recovered from skin tissues for up to 4 months, even after surface swabbing resulted in negative cultures, perhaps suggesting that such prolonged persistence could be attributed to its potential to survive deeper within the skin tissue.

The fungal factors contributing to strain or clade-specific advantages in skin colonization have only begun to be explored. Utilizing both an ex vivo human skin model and an in vivo murine epicutaneous model, we specifically investigated the role of adhesins in skin colonization in 2 clade I isolates, AR0382 and AR0387 [23]. We observed a noticeable difference in the fungal burden between these 2 closely related isolates. This variation in colonization capacity could be partly attributed to the differential expression of 2 adhesins: the canonical adhesin Iff4109 and the *C*. *auris*-specific adhesin Scf1 [23]. Notably, the deletion of *SCF1* and *IFF4109* in AR0382 led to a reduced ability to colonize both ex vivo human skin explants and in vivo murine skin. Conversely, overexpressing *SCF1* in AR0387 significantly enhanced its skin colonization capacity. Another report found increased bioburden in skin colonization models associated with strain-specific increased expression of the adhesin Als4112 [102]. Expression of these adhesins may directly mediate surface association with the skin substrate. As adhesin expression varies widely among *C*. *auris* isolates, this may substantially explain variability in strain colonization potentials [23,102]. Interestingly, a study using an intradermal infection model observed nearly equivalent fungal burdens among 4 strains from the 4 major clades, suggesting the source of strain-specific variation may have been bypassed by intradermal inoculation in this case [103]. Natural variation observed in colonization potential then likely predominantly reflects variation in skin surface association.

In humans, patients colonized with *C*. *auris* can remain positive for extensive periods of time [7,58]. The observation that *C*. *auris* can reside in the hair follicle and persist for months in murine colonization model may in part explain the prolonged colonization of *C*. *auris* on patient skin [32]. In addition, the recovery of *C*. *auris* cells from skin tissues even after negative cultures from surface swabs could be reflected by challenges in identifying *C*. *auris* colonization during patient screening. Proctor and colleagues reported that taking swab samples from at least 6 body sites, including nares, palm and fingertips, toe webs, perianal skin, inguinal crease, and axilla, maximizes the sensitivity of colonization screening [11]. However, for routine screening and practicality, the authors recommended focusing on high-yield areas such as the armpits, inguinal creases, and anterior nostrils [11]. Antiseptic agents such as chlorhexidine gluconate (CHG) are routinely utilized for skin care and decontamination in long-term care patients. Using an in vivo murine model, Huang and colleagues showed that treating mice with CHG prior to or post *C*. *auris* colonization significantly reduced fungal burden on the skin [32]. However, some reports suggest *C*. *auris* can continue to spread even after the introduction of unit-wide CHG bathing [7], indicating the effectiveness of CHG bathing in reducing *C*. *auris* skin colonization requires additional investigation.

Despite the advancements in our understanding of *C*. *auris* colonization dynamics, the ability of available colonization models to recapitulate human data is largely unexplored. First, it is unclear whether critical interactions between *C*. *auris* and the human skin microbiome would be recapitulated by the in vivo murine models. One study showed distinct microbiome compositions associated with healthy patients compared to *C*. *auris*-colonized patients [11]. The microbiome of *C*. *auris*-negative samples were dominated by *Malassezia* species and skin commensal bacteria species: *Staphylococcus hominis*, *Corynebacterium tuberculostearicum*, *Staphylococcus epidermidis*, *Staphylococcus caprae*, and *Corynebacterium striatum*. However, *C*. *auris*-positive samples were associated with various *Candida* species along with bacteria such as *Pseudomonas aeruginosa*, *Klebsiella pneumoniae*, *Providencia stuartii*, and *Proteus mirabilis*. Whether this association has a causal influence on *C*. *auris* colonization remains unclear. However, the authors proposed an example mechanism that could directly link the *C*. *auris*-associated microbiome with *C*. *auris* colonization, pointing to reports demonstrating that the skin commensal *S*. *epidermidis* induces the expression of the antimicrobial peptide LL-37 in human keratinocytes [104], which could, in turn, inhibit *C*. *auris* growth and skin colonization [105].

Furthermore, the physiological conditions present in distinct body sites favored by *C*. *auris* for skin colonization may not be fully captured in existing models. Common colonization sites like the axilla, groin, nostrils, and fingertips contribute to both persistent infections and transmission risks, although each body site presents a unique colonization environment [11,106]. For instance, differing from the epidermal cells found in other areas, the nares are lined with mucosal epithelium, which secretes mucus, contributing to the nares’ moist environment. Alternatively, the axillae are home to a higher concentration of sweat glands and typically exhibits a pH of 6.5, in contrast to the more acidic pH of 5.5 found in other skin areas [107]. This reduction in acidity is associated with altered bacterial growth, resulting in a distinct microbiome compared to other body sites [108]. Where *C*. *auris* robustly colonizes diverse skin niches, adaptations to distinct environmental pressures are likely to inform differential colonization dynamics.

### Antifungal resistance

The high rate of acquired antifungal resistance in *C*. *auris* poses a substantial threat to treatment efficacy. While antifungal susceptibility cutoff values have not been established for *C*. *auris*, the US CDC has proposed tentative breakpoints. Based on these values, it is estimated that 80% to 90% of isolates exhibit fluconazole resistance, 20% to 50% of isolates exhibit amphotericin B resistance, and 5% to 7% are resistant to echinocandins [5,109]. The rate of antifungal resistance in non-*auris Candida* species overall is estimated at only 7%, demonstrating remarkable plasticity and adaptation in *C*. *auris* by comparison [95]. Notably, resistance rates vary by clade, with isolates of clades I and III demonstrating almost universal resistance to fluconazole, isolates of clade II being widely susceptible, and isolates of clade IV demonstrating variable resistance [5]. These trends are largely associated with acquired and lineage-specific mutations in genes encoding drug targets or efflux regulators, highlighting stable adaptability in *C*. *auris* even around cellular processes with potentially impactful fitness implications. Multidrug and even pan-resistant isolates have demonstrated the capacity to spread and transmit between individuals, despite any fitness costs potentially associated with the development of high levels of resistance, and resistance is known to emerge upon therapy, limiting viable treatment options [110]. Specific mutations and mechanisms leading to acquired resistance have been extensively analyzed elsewhere, and we direct the reader to other excellent reviews for detailed discussion [95,109]. Briefly, characterized resistance mutations largely accumulate in genes encoding drug targets or regulators of efflux, in line with classical mechanisms of antifungal resistance. Somewhat enigmatically, however, *C*. *auris* also exhibits high rates of resistance to amphotericin B, a polyene antifungal rarely associated with resistance in other pathogenic fungi, and the vast majority of resistant isolates lack canonical resistance mutations [109,111] One recent report found experimentally evolved amphotericin B resistance most commonly emerged in association with membrane sterol modulation through mutation in *ERG* pathway genes, though high-level resistance frequently arose at the cost of growth rate or infection potential [112]. Using wild type isolates from 4 different clades, the authors observed strain-specific variability in both resistance development and resistance-associated fitness loss, suggesting the importance of differential genetic backgrounds for the emergence of acquired resistance. Mechanistically, the authors noted an evolved mutant with a compensatory mutation in the cAMP/PKA signaling pathway responsible for rescuing the fitness tradeoff associated with sterol modulation, and a similar mutation has been reported in 1 case of clinically acquired amphotericin B resistance [112,113]. This finding suggests a broader crosstalk between stress response and drug resistance, which may prove critical in understanding the development of antifungal resistance and variations in acquired resistance in divergent *C*. *auris* lineages.

## Aggregation

Filamentation and morphogenic plasticity are critical virulence traits in many fungal pathogens, including the model pathogenic yeast *Candida albicans*. While morphogenic transitions in *C*. *auris* are less apparent in response to canonical filamentation cues described in model species, numerous reports have documented cases of isolates exhibiting an alternative aggregative morphological state, with cells growing in multicellular conjoined structures. While some experimental evidence has suggested aggregation can influence host association [84,114,115], and while similar multicellular phenotypes have been argued to convey environmental advantages in nonpathogenic settings in other species [116,117], selective advantages for aggregation in *C*. *auris* remain largely speculative. Efforts to characterize this behavior suggests *C*. *auris* aggregation is the result of one of a multitude of phenotypes rather than a single phenomenon. Aggregative states have been reported as either a constitutive and heritable characteristic or as an inducible response to environmental conditions [114,118–120]. Some reports suggest aggregation as a phenotype is strain or clade specific, while others suggest any representative isolate from diverse clades can grow in aggregates [28,114,120]. Isolates from other *haemulonii* complex members exhibit similar heritable multicellular phenotypes, suggesting the evolution of aggregation is not specific to any *C*. *auris* lineage [121]. With aggregation being a largely qualitative phenotype with an observational but not always biological definition, the broad umbrella term of aggregation may encompass multiple molecular underpinnings. Aggregates may exhibit common selective advantages, such as those conferred by their physical multicellular structure, but may also exhibit distinct phenotypes specific to the molecular mechanisms associated with the aggregative state.

**Fig 3:**
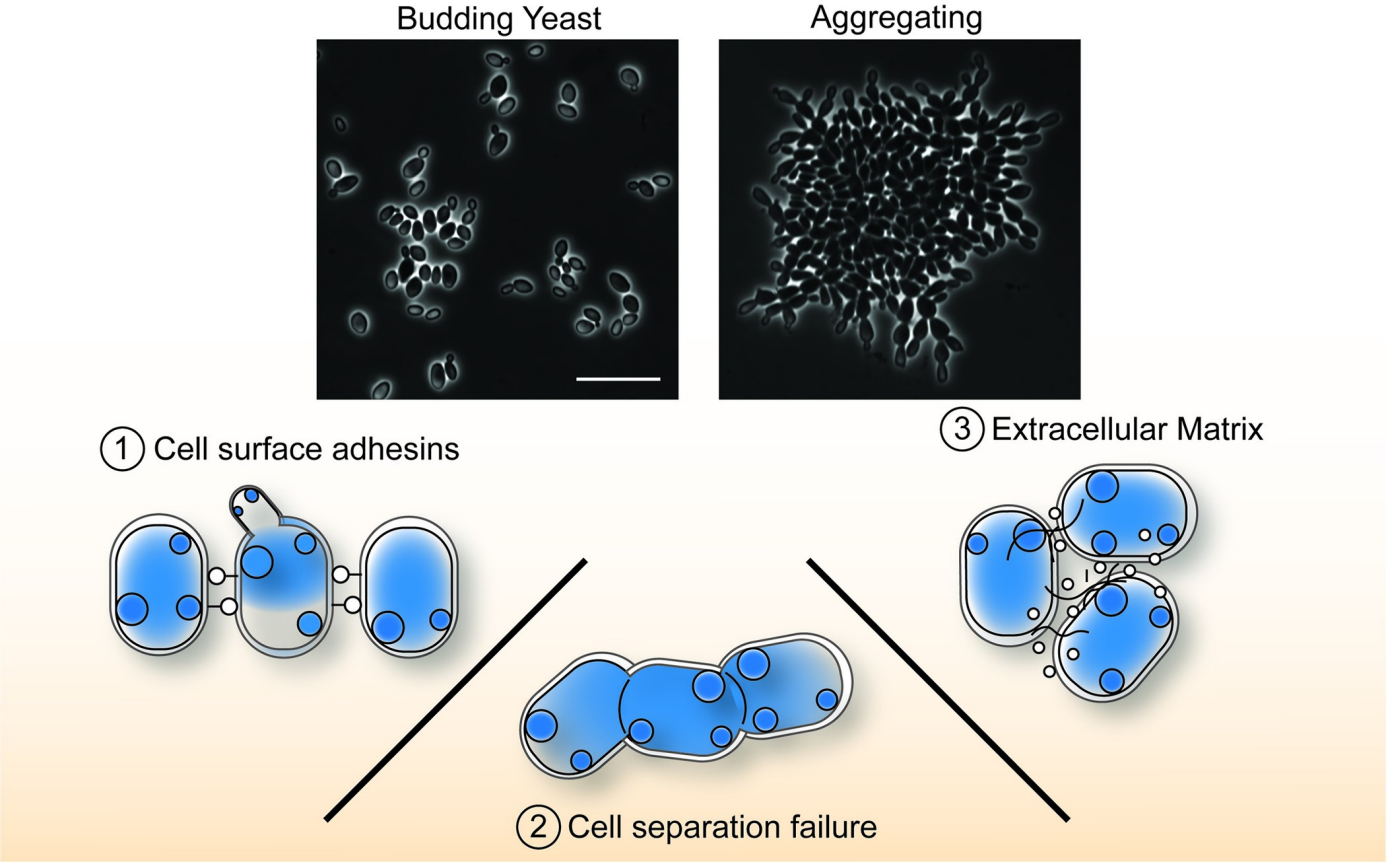
Three distinct molecular mechanisms of *C*. *auris* aggregation. *C*. *auris* can exist either in a unicellular, budding yeast morphology or in multicellular aggregates (phase contrast microscopy, top; scale bar = 20 μm). Three distinct mechanisms of aggregation have been reported: (1) Cell–cell adhesion mediated by cell surface–bound proteins, with strong evidence for a prominent role by the adhesin Als4112. (2) Failure of daughter cells to separate from parent cells after budding, often due to incomplete septum degradation. (3) Cohesive multicellular clusters conjoined by secreted extracellular components.

Three distinct mechanisms of aggregation have been described: (1) Adhesin-mediated aggregation; (2) Cell separation defects; and (3) Extracellular matrix aggregation (Fig 3).

Adhesin-mediated aggregation appears to be the result of cell–cell adhesion driven by cell surface proteins. For *C*. *auris*, the *ALS* family adhesin encoded by the reference locus B9J08_004112 (*ALS4112*) is frequently reported in association with cellular aggregation. In *C*. *albicans*, Als5 and other cell wall proteins promote cell–cell adhesion through the formation of amyloid aggregates [122,123]. The mechanism for Als4112 aggregation in *C*. *auris* may be similar, as treatment of aggregating isolates with amyloid-inhibiting compounds Thioflavin-T or Congo Red partially suppresses aggregation [120]. Interestingly, 1 report detailed an isolate exhibiting reversible aggregation when suspended in PBS that could be suppressed by suspension in pure water, which could potentially be explained by electrostatic requirements for amyloid formation [119]. Disruption of some forms of aggregation by Proteinase K also suggests a cell surface proteinaceous mechanism [102,119]. A similar mechanism may be responsible for aggregation in other *C*. *haemulonii* complex members, which can be disrupted by treatment with Proteinase K, trypsin, or SDS [121].

Transcriptional control of Als4112 specifically has been demonstrated as a mechanism for development of an aggregative state. Numerous reports have detailed aggregation in response to subinhibitory concentrations of triazoles and echinocandins [31,124,125]. One study found overexpression of *ALS4112* associated with aggregation in a strain grown for multiple generations under subinhibitory concentrations of caspofungin [124]. In this case, removal of the caspofungin stress resulted in loss of aggregation and reduced *ALS4112* expression. This behavior is reminiscent of *ALS*-mediated echinocandin-induced aggregation described in *C*. *albicans* [126]. In another case, Bing and colleagues identified naturally aggregating isolates that exhibited substantial copy number increases in the *ALS4112* locus [102]. Similarly, a clade III isolate that exhibited aggregation when grown in SabDEX, but not RPMI, demonstrated transcriptional overexpression of *ALS4112* under aggregation–growth conditions but failed to aggregate when *ALS4112* was deleted [119]. Interestingly, the media-inducible expression of *ALS4112* and associated aggregation was not observed in a clade I isolate [119], although in our recent work, we found that overexpression of *ALS4112* in a clade I isolate by promoter replacement was sufficient to drive aggregation, suggesting the strain-specific phenotypes reported in association with *ALS4112* aggregation are likely due to regulation of the gene rather than clade-specific adhesin sequence variation [23].

As an alternative mechanism of aggregate formation, isolates exhibiting defects in cell separation after budding have been described. Mechanistically, this can result from a failure of septum degradation. Through genetic screening, we identified aggregative mutants associated with defects in the Regulation of *ACE2* and Morphogenesis (RAM) pathway [115]. The RAM pathway is a conserved regulatory network that controls daughter cell localization of the terminal transcription factor Ace2, which, in turn, regulates transcription of a suite of septum-degrading enzymes, including chitinases and glucanases [127]. The aggregative phenotype associated with defects in *ACE2* or upstream members of the RAM pathway is conserved in other *Candida* species and *Saccharomyces* [128,129]. Interestingly, cultivation under conditions that favor multicellularity can select for spontaneous RAM pathway mutants in model species [116], which may explain some instances of aggregation in *C*. *auris*. One report identified 2 urinary tract *C*. *auris* patient isolates exhibiting rugose colony morphology and strong cellular aggregation associated with a nonsense mutation in *ACE2* that was heritable over 30 passages over the course of 6 months [118]. This spontaneous emergence of heritable and constitutive aggregation may be mechanistically similar to aggregative or elongated isolates recovered in rare events after passage of nonaggregative parent strains through animal hosts, though the genetic bases for these reports remain unexplored [130,131]. Other reports have detailed transcriptional down-regulation of chitinase genes in naturally aggregating isolates, but it is unclear whether this is responsible for the aggregative phenotype or whether this down-regulation is the result of defects in the RAM pathway [120,132]. Interestingly, 1 report suggested cell separation defects may also contribute to echinocandin-induced aggregation [119].

A third proposed mechanism for aggregation relies on extracellular matrix (ECM) connecting cells together. In this case, aggregation is the result of an inducible response associated with production of ECM. Through SEM, Malavia-Jones and colleagues observed aggregates connected by ECM in cells cultured at 37°C [120]. This inducible aggregation was observed in isolates from all 4 major clades, even for isolates that exhibited no aggregation or notable ECM production when cultured at 30°C [120]. This finding may be consistent with an observation that all isolates from a panel of 19 strains, representing aggregative and nonaggregative phenotypes in vitro from all 4 major clades, formed aggregates in infected organs in a murine model [28]. Importantly, these reports suggest all *C*. *auris* isolates have the potential for aggregation under specific conditions.

Morphological variation is a critical pathogenic feature in *C*. *albicans* and other related fungi, so it is reasonable to examine aggregation in *C*. *auris* from a clinical perspective. Aggregating isolates are often found to be less virulent in experimental models compared to nonaggregating isolates (for instance, [84,114,115]), although exceptions are common, with reports that strain-specific virulence differences are independent of aggregation (for instance, [25,28,119]). Importantly, quantitative comparisons in virulence models should be examined carefully, as standardization of infectious units in inocula between single-celled yeast and multicellular aggregates is unrealistic, and colony-forming unit recovery can be unreliable for aggregating isolates [28]. Still, physical or biological variation may meaningfully contribute to virulence. One report found that phagocytic uptake by THP-1 cells was minimal for aggregating isolates, and it has been suggested that aggregation may be a mechanism of immune evasion in vivo [75,119]. In the same experiment, stray single *C*. *auris* cells were efficiently recognized by the immune cells, suggesting the physical bulk of aggregates may hinder phagocytosis [119]. This model is difficult to reconcile as an explanation for differential virulence between aggregative and nonaggregative strains, though, given the propensity of even nonaggregative strains to form aggregates in vivo [28,75]. It has also been suggested that aggregation may hinder dissemination in vivo, but this idea is inconsistent with observations that both aggregating and nonaggregating isolates exhibit similar capacities for tissue invasion in murine models [28,120]. Differences in virulence, then, likely arise from strain-specific biological variation beyond aggregation in and of itself.

## Outbreak potential

Clonally distributed outbreaks in healthcare settings remain a substantial driver of public health and clinical concern for *C*. *auris*. While carriage by an index patient represents the most likely primary reservoir in these settings, viable *C*. *auris* cells are rapidly and extensively shed to surrounding areas, and colonization of inert substrates in the nearby clinical environment can provide a secondary reservoir to potentiate outbreak progression. Here, we highlight biological observations exploring the persistence of *C*. *auris* nosocomially on abiotic reservoirs and its recalcitrance to decontamination efforts in these environments.

### Disinfection resistance

While high level and sporicidal disinfectants have widely been observed to be effective against *C*. *auris*, other disinfectants demonstrate unreliable and often unpredictable efficacy. Recommendations from the CDC and other authorities particularly caution against the use of water-based quaternary ammonium compounds (QACs) [133,134]. This places an additional burden on infection prevention efforts, as QACs are used extensively in healthcare settings for disinfection of noncritical patient care items and surfaces. Accordingly, the Environmental Protection Agency established the List P Registry to validate disinfectants against *C*. *auris* specifically, as experimental data suggest even QACs with *C*. *albicans* claims can exhibit poor efficacy against *C*. *auris* [133]. While these observations might give the impression that *C*. *auris* is inherently less susceptible to killing by QACs than *C*. *albicans*, the reality appears to be more complex. Disinfectant efficacy testing suggests the susceptibility of *C*. *auris* is dependent on the genetic background of the isolate tested as well as the formulation of the QAC. For instance, compared to clades I, III, and IV isolates, the clade II isolate-type strain consistently shows increased susceptibility to killing by diverse QAC formulations [33,34], including to a benzalkonium chloride QAC that demonstrated poor efficacy against *C*. *albicans* [35]. Bacterial acquired resistance to QACs most often stems from efflux activity [135]. The strain-specific resistance exhibited by *C*. *auris* may follow similar principles. Dire and colleagues found that pulsing a *C*. *auris* isolate with a subinhibitory concentration of benzalkonium chloride for 15 days increased its MIC to the QAC 4-fold [136]. This increase was associated with increased rhodamine-6G efflux activity, suggesting increased efflux may be related to the increased QAC tolerance [136]. This finding suggests *C*. *auris* isolates with low susceptibility to QACs may be adapted to increased efflux activity. In practice, this adaptation may even occur upon exposure to subinhibitory concentrations of disinfectant in clinical settings.

While consensus guidelines generally recommend against QAC usage for *C*. *auris* disinfection, susceptibility data are more complicated, with evidence of both strain-specific tolerance to certain formulations and specific activity of different formulations against different isolates, often in unpredictable associations. For instance, 2 Kinzua disinfectants with QAC blends as active ingredients showed strong efficacy (approximately 4-log reduction or greater) against *C*. *albicans* and clades I and II isolates of *C*. *auris*, while exhibiting only minor efficacy against clades III and IV isolates [33]. Meanwhile, another QAC-based disinfectant exhibited strong efficacy against *C*. *albicans* and a clade I *C*. *auris* isolate, while isolates from clades II, III, and IV were largely nonsusceptible [33]. Combinatorial formulations of QACs with other disinfectant classes has shown promise of increased efficacy, such as a QAC/polyhexanide blend that prevented growth and demonstrated a >5-log reduction against a panel of isolates from 3 clades [137] or QAC disinfectants supplemented with varying concentrations of isopropanol or ethanol demonstrating consistent efficacy against diverse isolates [33,34]. Interestingly, some formulations highlight further strain specificity in susceptibility, such as a QAC disinfectant containing only 17.2% isopropanol only exhibiting strong efficacy against clade II and clade IV isolates, but not others [34]. With understanding of disinfectant resistance mechanisms being largely incomplete, explaining strain-specific susceptibility to different active ingredients and formulations remains challenging. Efflux potential represents a promising explanation, but lineage-specific physiological differences, such as cell surface and membrane profiles, may also be relevant, especially given the differential activity of diverse QAC chemistries.

Ultraviolet-C (UV-C) devices are increasingly used to supplement chemical disinfection regimes. While protocols and devices vary considerably, several reports have examined the disinfectant efficacy of UV-C treatment on *C*. *auris* isolates from diverse backgrounds. Again, clade II isolates are frequently reported to exhibit greater susceptibility to UV-C killing than isolates from other clades [138,139]. Still, sensitivity to UV-C varies widely among isolates, though increasing exposure time and reducing distance from the UV source can sometimes improve killing of less susceptible isolates [139–142]. Interestingly, several direct comparisons have found *C*. *albicans* sensitivity in line with clade II and other highly susceptible *C*. *auris* isolates, while resistant isolates exhibit greater resistance to UV-C than *C*. *albicans* [138,139,141]. This variation suggests some *C*. *auris* isolates exhibit an adaptation that is protective against UV killing, though it is unclear whether such an adaptation would be selected for by UV pressure. One possible resistance mechanism is cellular aggregation. Chatterjee and colleagues observed UV susceptibility among isolates was qualitatively associated with aggregation potential, where nonaggregative isolates were more commonly susceptible [143]. In this case, 2 aggregative clade III isolates exhibited very little susceptibility, and killing could not be improved by increased exposure time [143]. As is typical of UV disinfectant efficacy, killing is most effective when the cells are directly exposed to the light source [141,144]. It is perhaps possible that exposed cells in large aggregates absorb UV radiation and protect internal cells, offering a resistance strategy implicit to the physical multicellular structure in aggregates that may explain strain-specific UV susceptibility.

### Nosocomial persistence

The frequent association of *C*. *auris* with healthcare outbreaks is often accompanied by widespread persistence in clinical environments. Inert surfaces near colonized patients show associated high rates of positivity [7,12], and colonized objects have been linked to transmission events between individuals and outbreak persistence [13,14,45,145]. Spread and transmission through contact-independent mechanisms are also possible, as air dispersal of *C*. *auris* cells to patient-inaccessible areas has been observed [146]. The contaminated environment, in turn, likely serves as a secondary reservoir to potentiate transmission, and examples of patients acquiring *C*. *auris* infection after being moved to rooms previously inhabited by colonized individuals have been reported [147]. The ability of *C*. *auris* to tenaciously colonize surfaces further extends to invasive medical devices, and, like for other *Candida* pathogens, colonization of indwelling equipment poses a substantial risk for the development of invasive infection [8,148]. Considering these and similar observations, *C*. *auris* outbreaks are met with extensive infection prevention responses, more so than other *Candida* species and in similar force as for classically hospital-associated bacterial infectious agents [9,134,149]. Challenges in adhering to such responses are thought to contribute to the continuingly increasing rate of *C*. *auris* outbreaks [9].

**Fig 4:**
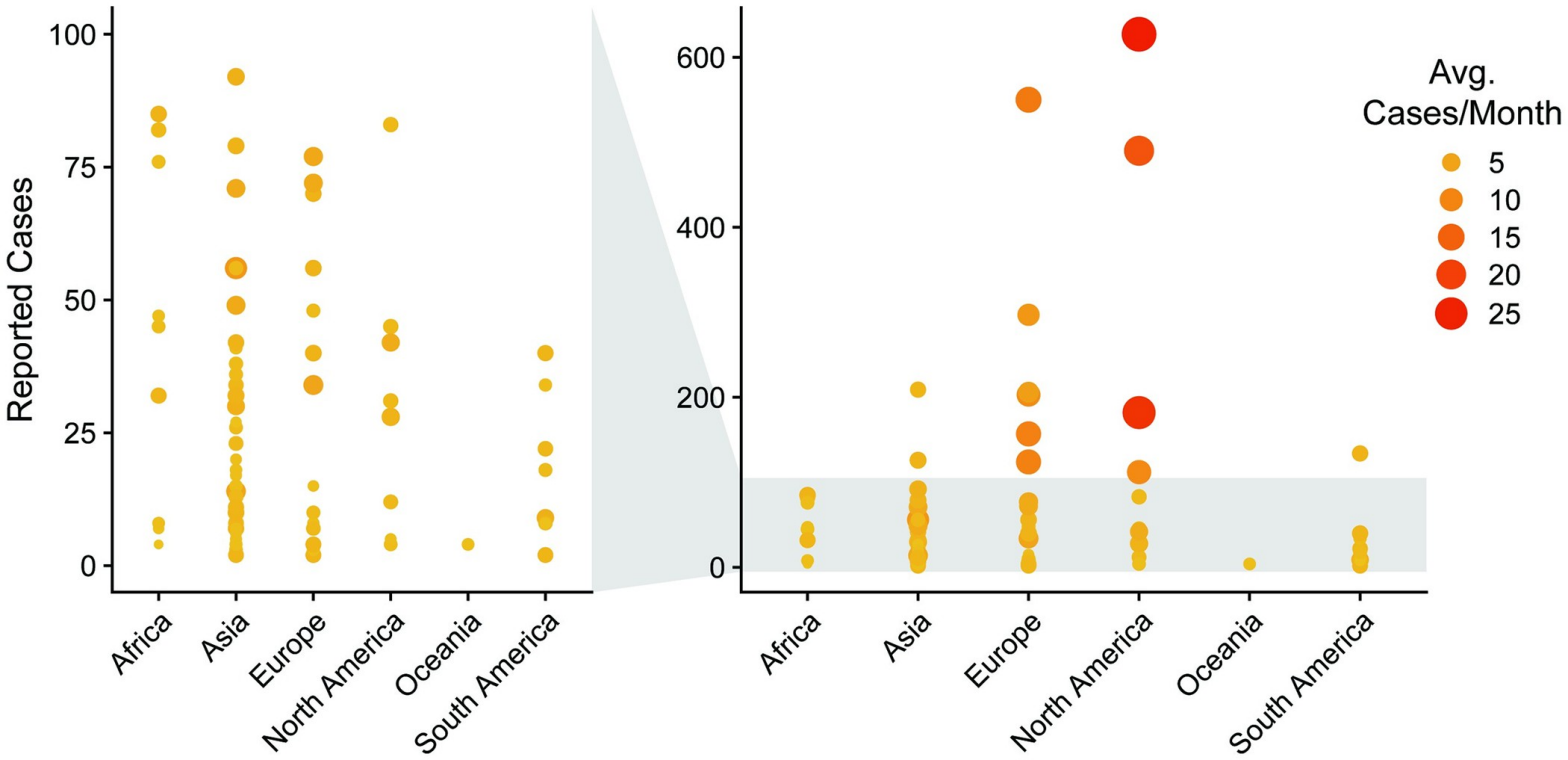
Size and transmission dynamics of single center or clustered multicenter *C*. *auris* outbreaks. Each point represents an individual outbreak report. Outbreaks are represented by total number of affected patients identified (colonized or infected) and by the rate of outbreak progression, defined by the average number of new cases identified per month. Inset (left panel) recapitulates all outbreaks comprising less than 100 affected individuals.

As is the theme of this review, we questioned whether this one-size-fits-all mentality fully captures the diversity of *C*. *auris* outbreaks. Examination of reports from single center or clustered multicenter outbreaks demonstrates widespread variability in both transmission rate and associated outbreak size, ranging from localized outbreaks involving single transmission events to major outbreaks involving rampant contamination of dozens of patients each month, leading to hundreds of affected individuals, with the caveat that variation in surveillance efficiency may be reflected in differences in reported outbreak sizes (Fig 4). Mapping of outbreak size and transmission rate failed to reveal obvious geographic associations between outbreak discrepancies, suggesting varying outbreak scenarios are possible despite differential infection control practices and recommendations from regional authorities.

In the most expansive outbreaks, hundreds of patients are affected across several years, resulting in near-endemic spread in multiple clustered facilities. For example, surveillance covering a 2.5-year period of a regional *C*. *auris* outbreak in the Chicago metropolitan area identified 490 clinical or colonized patients among multiple facilities, with within-facility prevalence as high as 71% [7]. Environmental sampling of an affected facility during 4 point prevalence surveys found 38% (73/191) of sampled objects and surfaces were colonized, even after routine cleaning and disinfection [7]. Contaminated objects included reusable and noninvasive patient care items, high-touch surfaces such as bedrails and doorknobs, and mobile objects such as nursing carts and mobile ultrasounds, providing a widespread reservoir for persistence and dissemination. Importantly, this example, as is representative of other large outbreaks, has largely been associated with long-term care facilities involving multiple-occupancy rooms filled with furnishings, care equipment, and personal items. These circumstances can promote persistent and prolonged colonization of patients as well as reduced opportunities for terminal and extensive room cleaning, likely substantially influencing outbreak progression.

A pattern of smaller outbreaks can also often be linked to *C*. *auris* persistence on abiotic substrates, demonstrating multiple acquisition events linked to a contaminated reservoir, but with much lower prevalence and transmission rates. One example of this is a single center observational study in the United Kingdom encompassing 2 general adult ICUs affected by a *C*. *auris* outbreak where a single index case lead to 6 transmission events over 6 months [13]. In this case, positivity was lower, with only 2.5% (6/236) of sampled objects collected over 3 environmental screens exhibiting *C*. *auris* contamination [13]. A single cloth lanyard (1 out of 100 sampled) attached to a controlled drug locker key was found to be contaminated, and removal of the cloth lanyards temporally correlated with termination of the outbreak [14,45,145]. Presumably, the contaminated lanyard represented a persistent reservoir, especially being a mobile object handled by numerous healthcare personnel and, importantly, not being subject to routine disinfection, leading to intermittent transmission events. This pattern may be common, as colonized medical devices such as temperature probes or surgical knives have similarly been linked to outbreak progression, and in some cases, exposure to colonized equipment has been demonstrated as an independent risk factor for development of colonization or infection [149].

Curiously, a contrasting scenario is not uncommon: a single index case with limited environmental persistence and no identified transmission events, despite high occupancy and room sharing. In 1 example, 1 patient demonstrating both skin and invasive *C*. *auris* culture was identified in an oncology ward at a New York hospital, prompting surveillance and deep hospital sampling over the course of 3 weeks [150]. In total, 48 samples from 18 patients and 132 samples from environmental surfaces throughout the patient room and ward were screened [150]. In this case, no other patients were affected, including one who shared a room with the index patient [150]. From environmental sampling, 2.3% (3/132) of samples demonstrated positivity, with 2 sequential positive samples taken between cleanings from a single reclining chair in the patient’s room [150]. Thirty other samples from the patient’s room were negative, as were the remaining samples from throughout the ward [150]. The stark contrast in colonization and dissemination rate in such examples raises the question as to whether the failure of *C*. *auris* to spread and persistently colonize the environment in these circumstances could be attributed to intrinsic and strain-specific colonization potential.

Efforts to describe colonization dynamics experimentally have largely focused on measuring persistence under desiccation and biofilm formation, and there is evidence in each case of differential behavior between strains. Welsh and colleagues measured the persistence of a clade I isolate dried onto a plastic surface and found recoverable CFU up to 14 days after inoculation and detectable esterase activity for up to 28 days [151]. Similar results were reported in a study that modeled surface contamination by suspending cells in PBS, artificial saliva, or fetal calf serum before drying cells on a plastic surface, in this case finding recoverable cells 14 days after inoculation [152]. The surface being colonized appears to be important for persistence as well; Dire and colleagues found that fabric, plastic, steel, and wood sustained colonizing cells for at least 21 days under wet or dry conditions, and at times even supported growth, while viability for wet or dry cells on glass decreased significantly by 14 or 21 days [152]. Another report observed survival of *C*. *auris* cells on latex or nitrile gloves up to 3 minutes but could not detect viable cells by 5 minutes [153]. These initial evaluations suggest survival for minutes to weeks in dry, nutrient poor conditions is plausible. Longer timeframes are likely; for instance, 1 clinical report identified a cloth lanyard that was potentially colonized for several months [151]. Careful quantitative comparison between related and unrelated isolates under consistent experimental conditions is needed, however, to understand the molecular basis for *C*. *auris* persistence on inert surfaces and to examine strain-specific adaptations. One report examined persistence of 2 distinct isolates after drying on a plastic surface [152]. While both isolates exhibited viability after 14 days, the recoverable CFU differed by 2 to 4 orders of magnitude depending on experimental conditions, suggesting strain-specific tolerance for desiccated survival [152].

As an alternative model for surface colonization and persistence, several studies have characterized the ability of *C*. *auris* to form biofilms. While, experimentally, biofilm-grown cells exhibit increased persistence and tolerance to decontamination [136,154,155], it is not well understood where *C*. *auris* would exist in a biofilm state during a nosocomial outbreak. Colonized sinks and catheters are likely to support biofilm growth given liquid flow and nutrient access, and experimental models suggest cells adopt biofilm characteristics when grown on skin, but individual cells transmitted to dry, nutrient poor surfaces are perhaps less likely to be found in biofilm states [8,71,98,156]. Several reports have demonstrated substantial quantifiable variability in biomass from biofilm formation between isolates [23,26,102,152,157,158]. Mechanistically, strain-specific variation in biofilm formation has been linked to differential expression of specific adhesins: either Scf1 or Iff4109, which mediate the attachment of cells to colonized surfaces, or Als4112, which mediates cell–cell attachment [23,102]. *SCF1* exhibits strain-specific transcriptional variation across isolates from every major clade, and differences in its expression are associated with differences in bioburden in colonization and biofilm formation on surfaces such as catheters or skin [23]. Expression variation in *ALS4112* may be common as well, as genotypic evidence suggests duplication and copy number variation around the *ALS4112* locus has occurred in numerous isolates, and associated overexpression correlates with colonization phenotypes [26,158,159]. Interestingly, some reports fail to find strain-specific variation in biofilm formation or colonization under certain experimental models, suggesting differential biofilm phenotypes may be the result of specific environmental conditions [26,159,160]. Understanding the appropriate experimental conditions to accurately model nosocomial colonization then will be key to accurately characterizing outbreak dynamics.

Beyond surface association, persistence, and biofilm formation, transmissibility from colonized substrates plays a major role in outbreak settings, but the mechanisms are still unclear. Strain-specific adhesin expression results in strains with reduced capacity for surface association and tenacity against shear force [23]. This phenotype may be associated with greater rates of dissemination from surfaces at the expense of reduced colonizing biomass. Similarly, in skin colonization models, different isolates produce varying bioburden [146], but the associated consequences on differential levels of shedding from colonized skin, transmission upon contact, or rates of environmental dissemination are unknown. *C*. *auris* has also been found to disperse across long distances through air transmission, but the dynamics of this mode of dissemination have not been explored [160–164]. Understanding the molecular mechanisms underpinning *C*. *auris* outbreak spread will require experimental modeling of transmission in addition to colonization and persistence and may ultimately yield critical insights into intrinsic fungal factors driving outbreak development.

## Conclusions

In this review, we have focused on virulence and other clinical phenotypic differences between strains and clades of *C*. *auris*, although there are many phenotypic differences that we have not discussed. It is likely that these differences in core biology will also impact the ability of *C*. *auris* to cause disease and transmit, and future work on the connections between genotype and phenotype in these different strains will be needed to fully understand this emerging pathogen.

From our analyses, we see a few critical points to consider for future research. It appears that clade IV isolates have a higher pathogenic potential than isolates from other clades; however, the molecular underpinnings of this are currently unknown. On the other hand, clade I isolates appear to tolerate acquisition of drug resistance mutations more than other clades. The relative impact of these 2 features on disease outcomes is still unknown. We also observe multiple different modes of aggregation, including some that are strain specific and others that appear to be generalizable across *C*. *auris*. The importance of these different modes of aggregation during infection still needs to be determined. Lastly, the potential strain-specific differences in outbreak potential is of critical importance, and in this case, we do not observe clade-level trends beyond the lack of outbreaks that can be attributed to clade II strains.

Going forward, it will be important to clarify which strain of *C*. *auris* is being investigated for each research question. The differences between strains also provide an opportunity to leverage comparative genomics approaches to map out specific genotypic variants associated with a phenotype of interest. Variation between strains within a clade provides an opportunity to understand specific evolutionary selective pressures. Importantly, this variation should be considered when deciding which strain is an appropriate wild-type strain for molecular analyses. Recent work in the model fungal pathogen *C*. *albicans* has identified that the reference SC5314 strain, in many cases, behaves differently than other isolates of *C*. *albicans* for clinically relevant phenotypes, including induction of host inflammation, degree of filamentation, and level of commensal colonization [161–165]. For *C*. *auris*, as a field, we have the opportunity to perform analyses across clades that will then allow us to define both species-level and strain-level differences and chose an appropriate strain for laboratory experiments to define mechanisms.
